# From steatosis to cirrhosis: the role of obesity in the progression of liver disease

**DOI:** 10.1007/s40200-025-01754-x

**Published:** 2025-10-14

**Authors:** Klaudia Nowak, Maria Paluch, Maja Cudzik, Klaudia Syska, Wiktoria Gawlikowska, Jakub Janczura

**Affiliations:** Collegium Medicum, Jan Kochanowski University, Kielce, Poland

**Keywords:** Obesity, Steatosis, Cirrhosis

## Abstract

Metabolic dysfunction-associated steatotic liver disease (MASLD), the most common subtype of steatotic liver disease (SLD), affects approximately 38% of the global adult population and is strongly linked to obesity, insulin resistance, and type 2 diabetes mellitus (T2DM). Projections estimate its prevalence may exceed 55% by 2040. Obesity plays a central role in the progression from simple steatosis to metabolic dysfunction-associated steatohepatitis (MASH), fibrosis, cirrhosis, and hepatocellular carcinoma. Excess adiposity contributes to hepatic fat accumulation, systemic inflammation, insulin resistance, and activation of hepatic stellate cells. key mechanisms in liver injury and fibrogenesis. Diagnosis traditionally relied on liver biopsy, but noninvasive techniques, along with serum-based indices, are now commonly used. MASLD is associated with an increased risk of cardiovascular disease, chronic kidney disease, and endocrine disorders, particularly in obese individuals. Management is centered on weight reduction, which can reverse steatosis, resolve MASH, and regress fibrosis depending on the degree of weight loss. Recent therapeutic advances include the approval of resmetirom, a thyroid hormone receptor-β agonist, and promising results from glucagon-like peptide 1 (GLP-1) receptor agonists. Bariatric surgery offers significant benefits in selected patients, improving liver histology and associated metabolic parameters. Despite these developments, no universally accepted pharmacotherapy exists for MASLD. Future directions include expanding access to diagnostic tools, validating novel biomarkers, and implementing public health strategies targeting obesity to prevent progression to end-stage liver disease.

## Introduction

**Fig. 1:**
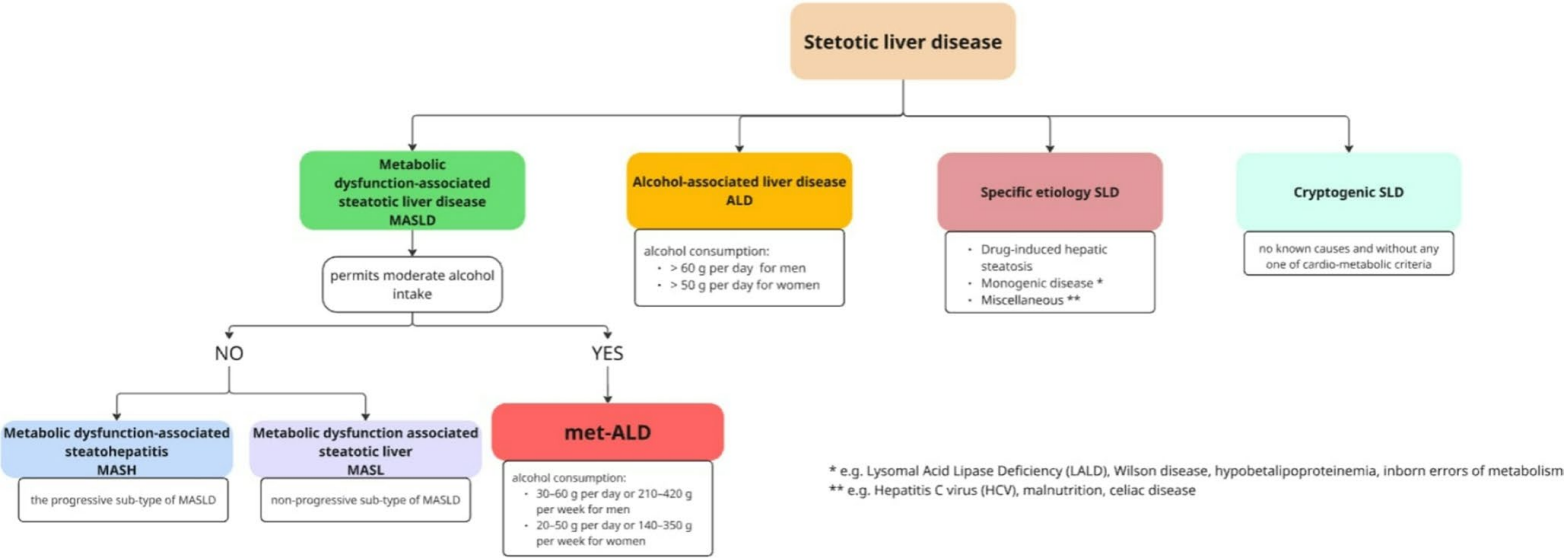
Classification of steatotic liver disease. Modified from reference [2]

**Fig. 2:**
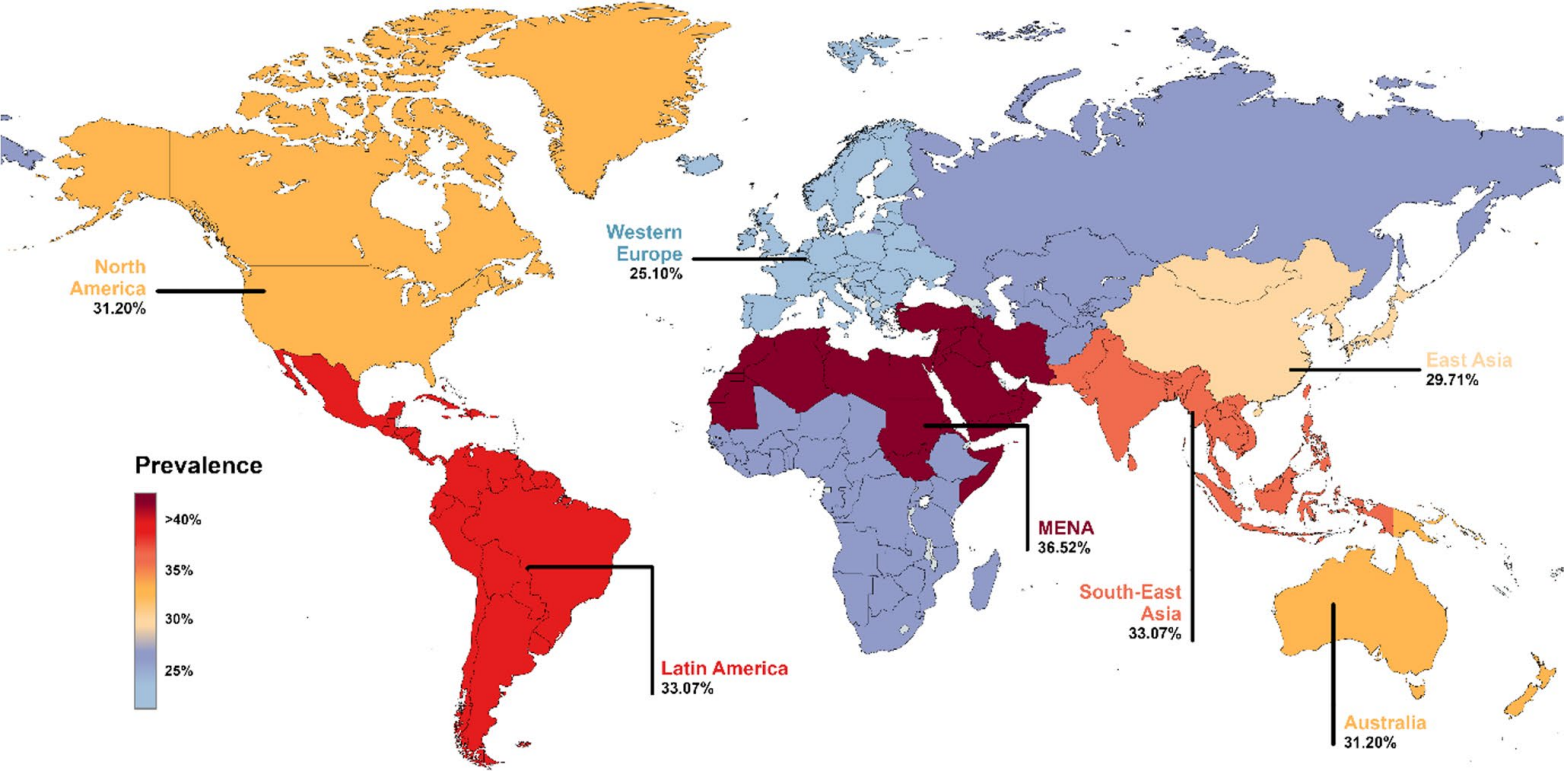
Prevalence of metabolic dysfunction-associated steatotic liver disease. Based on [3–6]

**Fig. 3:**
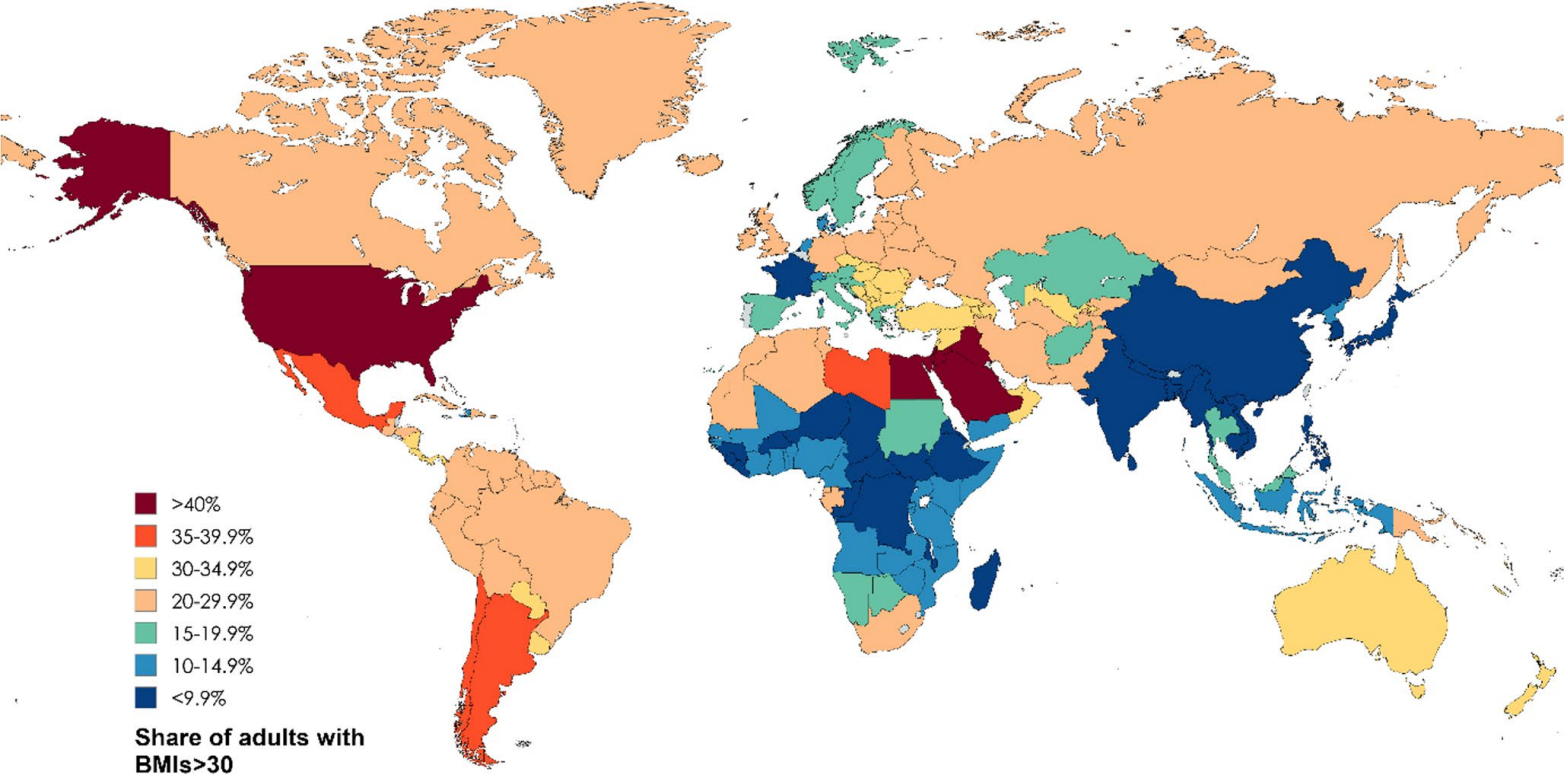
Share of adults with BMI >30, 2022. Based on reference [8]

**Fig. 4:**
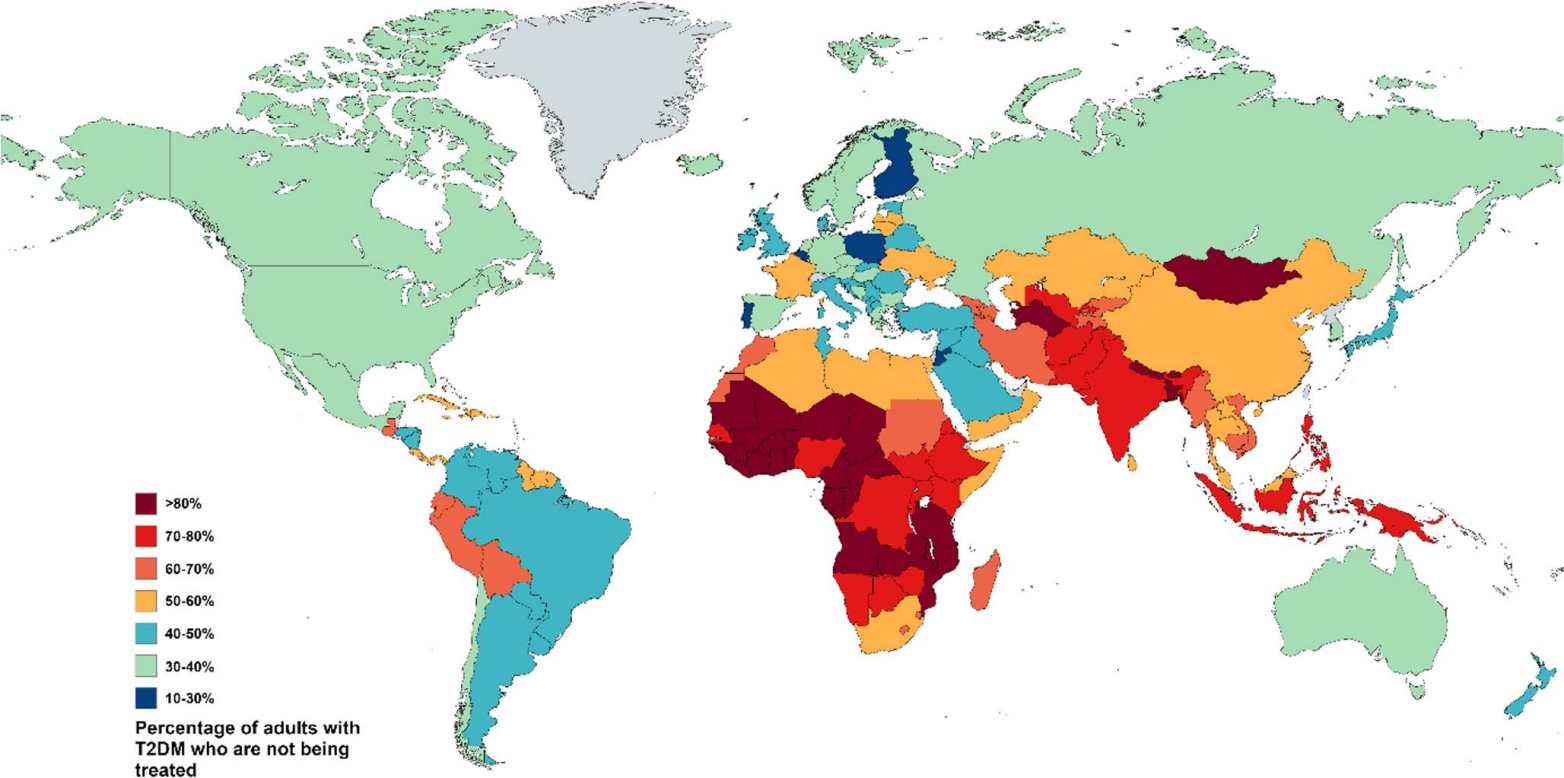
Global distribution of untreated T2DM, 2022. Based on reference [8]

Hepatic steatosis is a term describing abnormal lipids accumulation in hepatocytes exceeding 5%. In 2023, a Delphi consensus introduced a revision of the nomenclature for subtypes of Steatotic Liver Disease (SLD) [1]. According to the current classification system, the term SLD serves as an umbrella designation that includes a range of conditions leading to hepatic steatosis. These encompass metabolic dysfunction, alcohol-related liver damage, drug-induced liver injury, monogenic disorders, and other less common causes, as well as cases involving multiple contributing factors (Fig. 1) [1, 2]. The most common form of SLD, known as metabolic dysfunction-associated steatotic liver disease (MASLD), currently affects an estimated 38% of adults worldwide, with the highest prevalence reported in the Middle East and South America (Fig. 2) [3–6]. Projections suggest that by 2040, its prevalence could exceed 55% of the global population [3]. The rising prevalence of MASLD has been strongly linked to key risk factors such as the ongoing obesity epidemic, type 2 diabetes mellitus (T2DM), hypertension, and hyperlipidemia [7]. In a recent meta-analysis, which included data from 8,515,431 overweight and obese individuals across 22 countries, the prevalence of MASLD was found to be 69.99% among those who were overweight and rose to 75.27% among those classified as obese, underscoring the significant role of excess body weight as a key risk factor for liver disease [7]. Given that in some countries more than 40% of adults have a body mass index (BMI) greater than 30, the actual global prevalence of MASLD may be considerably underestimated (Fig. 3) [8]. Similarly, MASLD affects approximately 70% of individuals with T2DM, with its prevalence rising steadily in recent decades. Between 1990 and 2004, the estimated prevalence was 55.86%, increasing to 68.81% during 2016–2021 [9]. This upward trend is likely influenced by the persistently high rates of untreated T2DM, despite significant advancements in diabetes treatment, particularly the introduction of glucagon-like peptide-1 (GLP-1) receptor agonists (Fig. 4) [8, 10]. However, access to these newer therapies remains limited in many low- and middle-income countries due to high costs and restricted availability [10]. The progression of MASLD is typically slow, subtle, and asymptomatic, often leading to underdiagnosis, particularly in the absence of systematic screening programs. An estimated 10% to 30% of MASLD cases advance to metabolic dysfunction-associated steatohepatitis (MASH) and further to serious liver complications [3, 7]. This progression is strongly linked to the development of cirrhosis and significantly increases the risk of HCC [11]. SLD can also develop in the context of chronic viral hepatitis, where persistent hepatic inflammation leads to hepatocyte injury and impaired liver function, further contributing to its high prevalence in endemic regions. However, studies indicate that the presence of SLD generally does not impact the effectiveness of chronic hepatitis treatment [12–14]. In this review, we explore the role of obesity in the progression of liver disease, with a particular focus on the pathophysiological mechanisms driving the transition from steatosis to fibrosis and cirrhosis. We also discuss strategies for prevention, as well as current diagnostic tools, biomarkers, clinical implications, and associated comorbidities.

## Pathophysiology of obesity-related liver disease

The progression from obesity to liver disease involves a connection of metabolic, inflammatory, and hormonal disturbances. Central to this transition is insulin resistance, which disrupts glucose and lipid metabolism, leading to increased lipolysis, elevated circulating non-esterified fatty acids, and hepatic lipid accumulation [15]. Adipose tissue dysfunction, characterized by altered adipokine secretion, chronic inflammation, and tissue hypoxia, plays a key initiating role in the development of MASLD. This dysfunction is particularly pronounced in individuals with obesity, among whom MASLD affects between 50% and 90% individuals [16, 17]. These changes promote hepatic steatosis, lipotoxicity, and oxidative stress, which in turn activate pro-inflammatory and pro-fibrotic pathways driving the progression to MASH, fibrosis, and cirrhosis [16]. Lipotoxicity is a hallmark of obesity-associated liver disease. Excessive hepatic lipid accumulation impairs mitochondrial function and promotes the generation of reactive oxygen species (ROS), which exacerbate inflammation and hepatocyte injury [18].These damaging processes ultimately activate distinct cellular death pathways, drive fibrogenesis, and contribute to the progression toward hepatocellular carcinoma (HCC) [18]. Obesity itself is a well-established risk factor for HCC and has been independently linked to increased HCC-related mortality. A recent meta-analysis involving 1,599,453 individuals found that premorbid obesity was associated with a twofold increase in the risk of death from HCC, with the association particularly pronounced in men and Western populations [19]. Interestingly, the majority of hepatocellular triglycerides in MASLD are derived from non-esterified fatty acids released by adipose tissue, rather than from dietary intake, which further underscores the role of obesity and adipose tissue dysfunction in the pathogenesis of MASLD [16]. Sex differences significantly influence the expression and progression of MASLD. Men are more likely to develop the disease, which may seem counterintuitive given that the prevalence of overweight individuals is currently slightly higher among women (44%) than men (43%) [20]. Sex hormones play a significant role in the development and progression of MASLD. Testosterone is associated with an increased risk of developing MASLD, whereas estrogen appears to have a protective effect [12]. This protective role is mediated through estrogen receptor-α signaling, which helps reduce insulin resistance and suppress hepatic lipogenesis, thereby slowing liver disease progression [12]. However, after menopause, when estrogen levels decline, women who develop MASLD face a higher risk of progressing to advanced fibrosis compared to men [21]. The pathogenesis of obesity-related liver disease is multifactorial, and understanding these mechanisms is important for developing targeted interventions to prevent progression from steatosis to cirrhosis.

### From steatosis to steatohepatitis

The progression of SLD to more advanced stages is influenced by the multiple coexisting risk factors [22]. Prospective studies have shown that certain modifiable factors significantly increase the risk of fibrosis progression. Weight gain of more than 5 kg worsens insulin resistance, and increases hepatic fat accumulation over time, underscoring the strong association between excess body weight and liver disease advancement [3]. Notably, in the Million Women Study, the adjusted relative risk of developing liver cirrhosis rose by 1.3 for every 5-unit increase in BMI. Visceral obesity, in particular, has emerged as a critical determinant of disease severity [23]. Each additional metabolic abnormality compounds the risk of liver disease progression. In a large population-based cohort study of nearly 1,500 individuals, Julián et al. assessed liver stiffness. Authors found that 2.8% and 1.9% of the general population exceeded liver stiffness measurement (LSM) thresholds of ≥ 8.0 kPa and ≥ 9.2 kPa, respectively, markers of moderate to advanced fibrosis. These proportions were significantly higher among patients with MASLD (7.1% and 5%) and those with dysglycemia (6.2% and 4.7%) [24]. Interestingly, due to the protective effect of estrogens, women in procreational age had a lower risk of liver fibrosis progression [24]. Obstructive sleep apnea (OSA) is another important contributor to the development and progression of hepatic steatosis. OSA induces intermittent hypoxia followed by reoxygenation, a cycle that promotes hepatic steatosis through activation of the hypoxia-inducible factor 1 (HIF-1) pathway [25]. It also triggers inflammation via the accumulation of ROS and activation of the NF-κB signaling cascade. Moreover, OSA stimulates the sympathetic nervous system and initiates systemic inflammation and vascular endothelial dysfunction. These processes lead to increased platelet activation, insulin resistance, dyslipidemia, and the exacerbation of metabolic syndrome [25]. Chronic alcohol consumption further accelerates fibrogenesis by promoting persistent hepatic inflammation and the deposition of fibrous tissue [26]. A key mechanism in alcohol induced fibrosis progression is the activation of hepatic stellate cells (HSCs), which produce extracellular matrix components [26]. Alcohol-associated liver disease (ALD) is a major global health concern, responsible for approximately 5.9% of all annual deaths [27]. Notably, the combined prevalence of heavy alcohol consumption and obesity has risen significantly, from 1.8% (95% CI: 1.2%–3.1%) in 1999–2000 to 3.1% (95% CI: 2.7%–3.7%) in 2017–2020, representing a 72% increase over two decades [28]. This alarming trend suggests that individuals with obesity may face dual risks: liver damage driven by both metabolic dysfunction and excessive alcohol intake, underscoring the urgent need for targeted interventions in this vulnerable population.

## Diagnostic tools, biomarkers, and obesity-related indices

### Imaging and fibrosis assessment

For many years, liver biopsy was considered the gold standard for assessing the extent of hepatic fibrosis. However, due to its invasive nature and associated risk of complications, it is increasingly being replaced by noninvasive imaging techniques [29]. The main noninvasive modalities currently in clinical use include vibration-controlled transient elastography (VCTE), point shear wave elastography (pSWE), two-dimensional shear wave elastography (2D-SWE), and magnetic resonance elastography (MRE). During transient elastography, two key parameters are assessed: LSM and the controlled attenuation parameter (CAP) [30]. LSM is primarily used to evaluate the degree of liver fibrosis. It reflects the stiffness of hepatic tissue, which increases with fibrosis severity [31]. However, LSM values can also be influenced by other factors such as hepatic inflammation, cholestasis, and hepatic congestion, which may lead to overestimation of fibrosis if not properly considered [31]. CAP is used to quantify hepatic steatosis by measuring the attenuation of ultrasound signals through the liver. CAP values correlate with the amount of liver fat but can be affected by BMI and subcutaneous fat thickness [32]. The advantages, limitations, and availability of these methods are summarized in Table 1 [29]. Selvaraj et al., in a recent meta-analysis involving 14,609 patients, assessed the sensitivity and specificity of five imaging modalities: VCTE, pSWE, 2D-SWE, MRE, and conventional magnetic resonance imaging (MRI). Among these, MRE demonstrated the highest diagnostic accuracy, with both sensitivity and specificity exceeding 80% for the detection of advanced fibrosis and cirrhosis [29]. However, despite its superior performance, the high cost and limited availability of MRE restrict its widespread use in routine clinical practice [29]. The authors concluded that when elastography-based tests are successfully performed, all of them offer acceptable diagnostic accuracy for identifying advanced fibrosis and cirrhosis [29]. Interestingly, a 2023 study demonstrated that VCTE has prognostic value comparable to histological fibrosis staging in predicting major clinical outcomes, including mortality, HCC, liver transplantation, and cirrhosis decompensation [33].

**Table 1:** Types of elastography

Type	Principles	Advantages	Limitations	Availability
**VCTE**	Evaluates liver stiffness, whereby increased stiffness correlates with a higher probability of fibrosis	Particularly useful in distinguishing advanced fibrosis from minimal or absent fibrosis	Reduced diagnostic accuracy in cases of severe obesity	Widely available
**pSWE**	Enables point-specific measurement of tissue stiffness; faster propagation of the shear wave through tissue indicates greater fibrosis severity	Precise measurement at a specific site	Requiring a high degree of precision	Moderately available
**2D-SWE**	Represents an advancement of pSWE, providing a two-dimensional elasticity map of the liver	More comprehensive assessment	More complex	Less available
**MRE**	Combines MRI with low-frequency mechanical vibrations to generate an elastogram	Most accurate, the highest diagnostic performance	Expensive, longer procedure time	Limited availability

Abbreviations: *VCTE* Vibration-controlled transient elastography, *pSWE *Point shear wave elastography, *2D-SWE *Two-dimensional shear wave elastography, *MRE *Magnetic resonance elastography, *MRI* Magnetic resonance imaging

## Emerging serum biomarkers

As both MASLD and MASH are typically asymptomatic and often discovered incidentally, early diagnosis is crucial [34]. It highlights the need for simple, accessible serum biomarkers that can be utilized in primary care settings without specialist involvement, enabling timely intervention to stop disease progression. Common abnormalities include elevated levels of alanine aminotransferase (ALT), aspartate aminotransferase (AST), and gamma-glutamyl transferase (GGT). However, these markers do not consistently correlate with histological severity and should therefore not be solely relied upon. The SteatoTest offers improved diagnostic accuracy compared to ALT and GGT alone, but it is more costly and less widely available, and it does not quantify the extent of hepatic steatosis [35]. The non-alcoholic fatty liver disease (NAFLD) Liver Fat Score, which incorporates the presence of metabolic syndrome or T2DM, serum AST, the AST/ALT ratio, and fasting insulin levels, demonstrates high diagnostic accuracy. However, fasting insulin test is not routinely performed, which limits its practicality [35]. To assess advanced fibrosis or cirrhosis, the FIB-4 index is commonly used. It is calculated using patient age, ALT, AST, and platelet count. While it is a useful tool, its reliability is reduced in patients younger than 35 years or older than 65 years [35]. Other serum-based markers include the Enhanced Liver Fibrosis (ELFTM) test and the aspartate aminotransferase-to-platelet ratio index (APRI). Ongoing research is evaluating the sensitivity and specificity of these biomarkers for monitoring fibrosis progression. However, no single marker has yet proven accurate or reliable enough to detect stage F2 fibrosis with confidence [36]. Currently, novel serum biomarkers for hepatic steatosis are in development, but none have been broadly validated to date, highlighting the continued need for research in this area. There is a strong demand for a simple, accurate, and affordable biomarker that could be used not only for early diagnosis but also for monitoring treatment response and long-term outcomes [35].

## Novel obesity-related indices

BMI is one of the most commonly used indicators for assessing obesity. However, it has several important limitations, it does not differentiate between fat and muscle mass, nor does it account for fat distribution, which are critical factors in evaluating liver health [37]. While BMI remains a convenient and widely applied tool, its accuracy is limited. As a result, alternative obesity-related indicators have been developed to provide a more precise assessment of hepatic steatosis. A brief comparison of these novel markers is presented in Table 2 [37]. Among the novel obesity-related indices, the Fatty Liver Index (FLI) demonstrated the highest area under the curve (AUC), indicating the strongest diagnostic accuracy. However, the authors also highlighted that FLI, along with the Lipid Accumulation Product (LAP) and the Hepatic Steatosis Index (HSI), all possess high predictive value and can serve as simple, cost-effective tools for MASLD screening in clinical practice [37].

**Table 2:** Novel obesity-related indices

Index	Description	Parameters Considered	AUC	Cut-off	Advantages	Remarks
CMI	Combines fat distribution with lipid profile	WHtR, TG/HDL ratio	0.869	0.543	Strong correlation with insulin resistance and cardiovascular diseases	More effective in women and younger individuals
VAI	Predicts the amount of visceral fat	WC, BMI, HDL, TG	0.832	1.475	Assesses visceral obesity	More accurate in European populations and healthy individuals; less accurate in Chinese population
FLI	Used to assess hepatic steatosis without imaging methods	BMI, GGT, WC, TG	0.912	22.97	Very high sensitivity and specificity	Particularly effective in groups: <35 years old, women, individuals with BMI < 25.
LAP	Reflects visceral lipid accumulation	WC, TG	0.894	26.07	Second most accurate index	Particularly effective in detecting SLD in non-obese individuals

Abbreviations: *AUC *Area Under the Curve, *BMI* Body Mass Index, *CMI* Cardiometabolic Index, *FLI *Fatty Liver Index, *GGT* Gamma-glutamyl Transferase, *HDL *High Density Lipoprotein, *LAP* Lipid Accumulation Product, *MAFLD* Metabolic-Associated Fatty Liver Disease, *TG* Triglicerides, *VAI* Visceral Fat Index, *WC *Waist Circumference, *WHtR* Waist-to-Height Ratio

### Clinical implications and comorbidities

SLD is a multisystem condition that extends beyond hepatology. It represents the hepatic manifestation of metabolic syndrome and has far-reaching clinical implications, influencing the course of various chronic diseases. SLD most commonly affects the cardiovascular system, T2DM, chronic kidney disease (CKD), and other metabolic and endocrine disorders [38]. Obesity is a key driver of SLD and plays a central role in its systemic consequences. Excess adiposity, particularly visceral fat, contributes to insulin resistance, dyslipidemia, and chronic low-grade inflammation, all of which are shared mechanisms linking obesity to multi-organ dysfunction. The most significant clinical consequence of SLD is an increased risk of cardiovascular disease [38]. Cardiovascular conditions are the leading cause of death in patients with MASLD. A meta-analysis involving over 34,000 patients reported that MASLD increases the risk of both fatal and non-fatal cardiovascular events by 64% [39]. The underlying mechanisms include chronic systemic inflammation, hepatic insulin resistance with increased hepatic glucose output, endothelial dysfunction, and atherogenic dyslipidemia. In SLD, this dyslipidemia is characterized by elevated triglycerides and very-low-density lipoproteins (VLDL), along with reduced levels of high-density lipoprotein (HDL) cholesterol, resulting in a lipid profile that promotes atherosclerosis [40]. These abnormalities are often more pronounced in individuals with obesity, which further amplifies cardiovascular risk. Patients with SLD are also more susceptible to both supraventricular and ventricular arrhythmias. In a 10-year follow-up study of 400 patients with T2DM, those with coexisting MASLD had a significantly higher incidence of atrial fibrillation compared to those without MASLD (*P* < 0.005) [41]. SLD also significantly impacts renal health by contributing to the development of CKD, with the risk of kidney dysfunction increasing alongside the degree of liver fibrosis. In a large cohort study, the risk of incident CKD rose progressively with fibrosis stage. (HR = 1.36 for F2, 1.67 for F3, and 2.22 for F4 compared to F0-F1) [40]. This relationship is driven by persistent inflammation, metabolic dysregulation, and vascular impairment, making it essential to closely monitor at-risk individuals for signs of nephropathy [40]. Individuals with obesity may face an even greater risk of renal complications when SLD is present. Obesity alone is a well-established risk factor for both the onset and progression of CKD, a relationship supported by characteristic histopathological changes [42].This underscores the importance of prioritizing this population in screening efforts, not only for liver disease but also for associated renal impairment, to enable early detection and intervention. Furthermore, the presence of MASLD doubles the risk of developing T2DM and complicates its management. A meta-analysis confirmed this association, showing that individuals with MASLD had a 2.2-fold higher risk of developing T2DM compared to those without the liver disease (*P* < 0.001) [38]. Patients with both T2DM and MASLD are at greater risk of cardiovascular complications and often experience more difficulty in achieving glycemic control, emphasizing the need for individualized treatment strategies that address both lipid metabolism and body weight [38]. In practical terms, a multidisciplinary approach to obesity-related liver disease is essential. Obesity not only drives the development of MASLD, but also accelerates the progression of its complications, including MASH, which in turn exacerbate other comorbidities. Addressing obesity is therefore a fundamental component in the prevention and management of SLD and its systemic consequences, and should be considered a clinical priority. The most common comorbidities observed in patients with MASLD are summarized in Table 3 [38]^,^ [40]^,^ [43].

**Table 3:** Summary of the most common comorbidities observed in patients with MASLD

Comorbid Condition	Estimated Prevalence (%)	Clinical Significance
Type 2 diabetes	40–70%	Worsens glycemic control and overall disease progression
Hypertension	50–70%	Increases the risk of stroke and heart failure
Dyslipidemia	60–90%	Leads to an atherogenic lipid profile
Cardiovascular disease	40–60%	Raises risk of fatal and non-fatal cardiovascular events by 64%; leading cause of death in MASLD
Chronic kidney disease	20–30%	Risk increases with liver fibrosis severity
Sleep apnea syndrome	25–80%	Linked to obesity and hypoxia

Abbreviations: *MASLD * Metabolic Dysfunction-Associated Steatotic Liver Disease

## Therapeutic approaches and lifestyle interventions

Therapeutic options for MASH include lifestyle modification, pharmacological treatment, bariatric surgery, and, in advanced stages, liver transplantation [44]. Lifestyle change remains the cornerstone of MASLD and MASH management. Weight reduction, regardless of the method used, has been shown to improve liver health: a 3–5% reduction leads to decreased steatosis, a 7% loss can result in regression of steatohepatitis, and a 10% loss is associated with fibrosis reversal [44]. Importantly, patients with MASH are at increased risk of developing sarcopenia, so a protein-rich diet combined with resistance training is recommended, as sarcopenia can worsen the severity of MASH and accelerate its progression to cirrhosis [44]. Dietary interventions such as low-fat, low-carbohydrate, Mediterranean-style diets and intermittent fasting have shown liver-related benefits. However, the Mediterranean diet appears to be more effective in reducing hepatic steatosis and maintaining cardiometabolic health compared to low-carbohydrate and low-fat diets. In addition, it appears to be easier to maintain for many years [3]. In a meta-analysis by Kawaguchi et al., following a Mediterranean diet in patients with MASLD contributed to a significant reduction in FLI compared to a control group that received no intervention or followed another type of diet (*P* = 0.019) [45]. In contrast, the evidence for the efficacy of intermittent fasting in MASLD is controversial, but some studies report that it contributes to statistically significantly reductions in LSM [46]. Pharmacological treatment has emerged as a promising strategy in the management of MASH, complementing lifestyle interventions. In 2024, resmetirom, an oral selective thyroid hormone receptor-β (THR-β) agonist, was approved as the first medication specifically for the treatment of MASH in patients without cirrhosis but with moderate to advanced fibrosis [47]. This marked a significant milestone in the therapeutic landscape of steatohepatitis. In a phase 3 randomized controlled trial involving 966 patients, NASH resolution without worsening of fibrosis was achieved in 25.9% of patients receiving 80 mg of resmetirom and in 29.9% of those receiving 100 mg, compared to only 9.7% in the placebo group (*P* < 0.001 for both comparisons). Additionally, resmetirom demonstrated a favorable effect on lipid metabolism. LDL cholesterol levels were reduced by 13.6% and 16.3% in the 80 mg and 100 mg groups, respectively, while the placebo group showed a negligible change of 0.1% (*P* < 0.001 for both comparisons). These findings confirmed that both doses of resmetirom were significantly more effective than placebo in achieving NASH resolution and improving liver fibrosis by at least one stage [48]. While resmetirom has been approved for the treatment of non-cirrhotic MASH, a substantial proportion of obese patients already present with advanced liver disease, including cirrhosis, for which no approved pharmacologic therapy currently exists. However, emerging evidence offers hope. The ongoing phase 2b SYMMETRY trial evaluated the efficacy of efruxifermin, a fibroblast growth factor 21 (FGF-21) analog, in patients with biopsy-confirmed, compensated cirrhosis (Child-Pugh A) due to MASH [49].The trial enrolled 181 adults, 80% of whom had both obesity and T2DM. Participants were randomized to receive either 28 mg or 50 mg of efruxifermin or placebo. The mean BMI was 36.1 in the 28 mg group, 34.5 in the 50 mg group, and 36.7 in the placebo group. After 96 weeks, the difference in fibrosis regression between the placebo and 28 mg group was 10% points (11% vs. 21%), and 16% points in the 50 mg group (11% vs. 29%). Furthermore, MASH resolution at 96 weeks was observed in 13% of the placebo group compared to 42% in those receiving efruxifermin [49].These findings suggest that efruxifermin may represent a promising future therapeutic option for patients with obesity-related cirrhotic MASH, a group for whom treatment options are currently lacking. MASLD affects approximately 57.5% of obese individuals, yet no widely accepted pharmacotherapy is currently available. Among the agents under investigation, GLP-1 receptor agonists have shown potential benefits [10]. Originally developed for obesity and T2DM, GLP-1 receptor agonists, such as subcutaneous semaglutide, have demonstrated improvements in liver health [10]. A study evaluating the efficacy of subcutaneous semaglutide over a 52-week treatment period demonstrated a reduction in MASLD severity by at least one grade on ultrasound. Additionally, liver fibrosis was reduced, likely due to decreased activation of hepatic stellate cells [10]. Thiazolidinediones also show therapeutic promise, producing a 47% reduction in liver fat and reducing both subcutaneous and visceral fat, which is especially beneficial in obese patients. These agents also contribute to lower AST and ALT levels [10]. Bariatric surgery may also be an effective intervention for improving MASH and liver fibrosis. Current guidelines recommend considering bariatric procedures in patients with MASLD/MASH who do not have cirrhosis [44]. Two meta-analyses assessing the efficacy of Roux-en-Y gastric bypass reported improvement in steatohepatitis. However, only about 30% of patients experienced fibrosis improvement following the procedure [3]. In a study of 180 patients with class II obesity and MASH, five-year outcomes showed MASH resolution in 66% of those who underwent Roux-en-Y gastric bypass, compared to 12% after sleeve gastrectomy and 22% after gastric banding [3]. These findings confirm the effectiveness of bariatric surgery as a therapeutic option for selected patients. These therapeutic strategies underscore the critical role of addressing obesity as the central driver of MASH, emphasizing the need for personalized, multi-modal interventions that target both excess weight and liver-specific pathology to effectively slow or reverse disease progression.

##  Conclusions and future perspectives

Obesity plays a central role in the pathogenesis and progression of SLD, particularly in driving the transition from MASLD to MASH, fibrosis, and ultimately cirrhosis. The global rise in obesity and metabolic syndrome has made MASLD the most common chronic liver conditions worldwide. Effective management requires a multidisciplinary approach that targets not only hepatic outcomes but also systemic metabolic health. Early diagnosis through non-invasive tools and serum biomarkers, combined with individualized lifestyle interventions and emerging pharmacotherapies such as resmetirom and GLP-1 receptor agonists, offer promising avenues for disease control. Looking ahead, future research should focus on the development and validation of accessible, cost-effective diagnostic biomarkers suitable for primary care, as well as expanding therapeutic options tailored to obese populations. Greater attention is also needed for public health strategies that address obesity prevention and improve access to treatment in resource-limited settings. Tackling obesity remains a cornerstone in curbing the global burden of MASLD and preventing its progression to end-stage liver disease.

## Data Availability

No datasets were generated or analysed during the current study.
